# Inflammation in stroke: initial CRP levels can predict poor outcomes in endovascularly treated stroke patients

**DOI:** 10.3389/fneur.2023.1167549

**Published:** 2023-06-09

**Authors:** Tom Finck, Philipp Sperl, Moritz Hernandez-Petzsche, Tobias Boeckh-Behrens, Christian Maegerlein, Silke Wunderlich, Claus Zimmer, Jan Kirschke, Maria Berndt

**Affiliations:** ^1^Department of Diagnostic and Interventional Neuroradiology, School of Medicine, Klinikum Rechts der Isar, Technical University of Munich, Munich, Germany; ^2^Department of Neurology, School of Medicine, Klinikum Rechts der Isar, Technical University of Munich, Munich, Germany

**Keywords:** inflammation, stroke, thrombectomy, C-reactive protein, stroke outcome, neuroinflammation

## Abstract

**Background and purpose:**

Inflammation has been linked to poor prognoses in cardio- and cerebrovascular conditions. As it is known to increase after ischemia, C-reactive protein (CRP) may serve as a surrogate for systemic inflammation and thus be a hallmark of increased tissue vulnerability. The question arises whether CRP in the acute phase of ischemic stroke, prior to mechanical thrombectomy (MT), might help predict outcomes.

**Materials and methods:**

A single-center collective of patients with large-vessel occlusion, who were treated via MT, was analyzed in this observational case–control study. Univariate and multivariate models were designed to test the prognostic value of inflammatory markers (CRP and leukocytosis) in predicting clinical outcomes (modified Rankin score >2) and all-cause mortality 90 days after MT.

**Results:**

A total of 676 ischemic stroke patients treated with MT were included. Of these, 313 (46.3%) showed elevated CRP levels (≥5 mg/l) on admission. Poor clinical outcome and mortality at 90 days occurred in 113 (16.7%) and 335 (49.6%) patients and significantly more frequently when initial CRP levels were elevated [213 (64.5%) vs. 122 (42.1%), *p* < 0.0001, and 79 (25.2%) vs. 34 (9.4%), *p* < 0.0001, respectively]. CRP levels were highly predictive for impaired outcomes, especially in patients with atrial fibrillation, in both univariate and multivariate models. Interestingly, patients with initially elevated CRP levels also showed more pronounced increases in CRP post-MT.

**Conclusion:**

Poor outcome and death occur significantly more often in stroke patients with elevated CRP levels before MT. Our findings suggest that stroke patients with atrial fibrillation and elevated inflammatory markers are of particular risk for poor outcomes.

## Introduction

Outcomes in ischemic stroke have improved greatly over the last decades in large part due to the wider availability of specialized stroke units as well as the establishment of endovascular therapy as the standard of care for patients who present with acute large-vessel occlusions (LVOs) (1–5). In recent years, the indication for mechanical thrombectomy (MT) has been extended, e.g., to later time windows and smaller vessels, with procedural techniques being continuously improved (6, 7).

Nonetheless, ischemic stroke continues to pose a significant burden as it remains a main cause of morbidity and mortality worldwide and accounts for a large proportion of quality-adjusted life years lost (8). Many multiparametric models for predicting good clinical outcomes after endovascularly treated stroke include obvious confounders, such as time to treatment or the success of revascularization. However, despite successful reperfusion in good time windows, the long-term clinical trajectory is not always as favorable as expected. Some mechanisms conveying adverse outcomes remain ill-explained, especially as complex markers such as neuronal damage serum proteins may also play an important and yet poorly understood role in influencing outcomes (9, 10).

Pro-inflammatory systemic environments have recently been identified to independently predict poor outcomes in a panoply of conditions, from ischemic heart disease to carotid atherosclerosis progression, as well as ischemic stroke treated via intravenous thrombolysis (11–15). Moreover, the more delayed cellular inflammation conveyed by leukocytosis has been shown to correlate with poor outcome after MT (16). All these observations are hinting at the role that neuro-inflammation could have in post-ischemic brain remodeling.

High-sensitivity C-reactive protein (CRP) is an accessible serum protein known to be a reliable marker for systemic inflammation but also mediate pro-inflammatory downstream cascades, making it a suitable candidate to assess the level of intra-individual inflammation (17, 18). Moreover, the observation that most stroke patients showcase high-CRP levels is noteworthy given that activation of the secondary complement system and subsequent secondary brain damage through CRP has been shown experimentally (19).

It remains unclear whether CRP is elevated in response to ischemia-induced neuronal damage or whether pro-inflammatory mechanisms themselves are causing a time-delayed injury of ischemic brain tissue, analogous to phenomena seen after thermic tissue shock (20). As inflammatory markers have been associated with atrial fibrillation and thromboembolic complications, it may also be conceivable that inflammation influences the pathogenesis of ischemic stroke (21).

Our assumption is that CRP levels in the very-early stage of ischemic stroke could be a hallmark of systemic inflammation and increase the *ad hoc* vulnerability of the brain tissue to ischemic stress as well as promote subsequent neuronal damage through mediation of a post-ischemic ischemia-inflammation cascade. Identification of this association would add to current knowledge in stroke care, identify patients at risk benefitting from the most intensified and early anti-inflammatory therapy regimes, and advocate for future interventional studies (22).

To address those questions, the present study aims to investigate an association of inflammatory blood markers (CRP and leukocytosis) with neurological outcome and mortality in a large cohort of ischemic stroke patients with LVO treated with MT.

## Methods

### Study design

Prospectively collected clinical, interventional, and outcome parameters of a large collective of endovascular treated stroke patients in a comprehensive stroke center were analyzed for this retrospective single-center case–control study. The association of inflammatory markers with outcome parameters was tested as outcomes.

The study was approved by the local ethics committee, and the need for patient consent was waived. The observational, retrospective study design did not interfere with the routine clinical workflow and did not influence therapeutic decision-making. The results were reported in adherence to the STROBE statement guidelines.

### Study population

This retrospective, single-center study included all consecutive patients with ischemic stroke due to LVO who were admitted at a single comprehensive stroke center and treated by MT between January 2017 and March 2021 (*n* = 1,012).

Patients with an unclear onset of symptoms (wake-up stroke) or a concurring cause of infection at both admission or during the first 7 days of hospital stay were excluded. Furthermore, we only included patients for whom inflammatory serum markers on admission and outcome data at 90-day follow-up were available.

The prospectively collected clinical and imaging data were retrospectively analyzed. Basic demographic, clinical, and interventional data of patients were gathered. NIHSS-certified neurologists assessed the National Institutes of Health Stroke Scale (NIHSS) score at the time of admission and discharge. Substantial neurological improvement (SNI) was defined as the difference between admission and discharge, with an NIHSS score of ≥8 or a discharge NIHSS score of ≤ 1.

The mRS score was used to measure disability premorbid at discharge and at follow-up, while a poor clinical outcome in the 90-day follow-up was defined as mRS > 2.

Outcome values such as mRS and mortality in the follow-up were assessed either through routine follow-up in-house visits or, if the patient was not present for various reasons, by phone call through an experienced study nurse.

Stroke pathogeneses were determined according to the international TOAST (Trial of ORG 10172 in Acute Stroke Treatment) classification based on diagnostic and clinical information available for each patient (23). Further variables that are relevant for the present study included the presence of atrial fibrillation as well as documentation of cardiovascular risk factors such as active smoking, hypertension, hyperlipidemia, and diabetes mellitus. Pre-existing treatment with platelet-inhibiting drugs or lipid-lowering therapies was documented. Administration of pre-interventional intravenous tissue-type plasminogen activator (tPA) thrombolysis was assessed. At the end of the endovascular procedure, successful recanalization was defined as mTICI 2b-3 (24). Time of symptom onset, time of admission, time of reperfusion, and corresponding procedure times were taken from the existing database. Time to admission was defined between symptom onset and admission. Reperfusion time was defined between symptom onset and mTICI ≥ 2b. In cases, when recanalization was not successful (TICI < 2b), the control series after the last maneuver was used as the time endpoint.

### Assessment of inflammation markers

In patients with elevated CRP levels (defined as >5 mg/l), hospital records were retrospectively screened to determine whether any cause of infection was apparent prior to stroke onset or became uncovered during the acute-care hospital stay.

Levels of CRP and leukocyte counts were determined from the emergency blood panel taken upon referral, prior to MT, as well as blood panels drawn within the first 7 days after MT. This allowed for the analysis of three models: (i) outcome prediction based on the level of inflammatory markers at the time of admission, (ii) subgroup analysis in patients for whom serum inflammatory markers were available in the hyper-acute phase (< 4 h after symptom onset), with an aim to exclude the effects of CRP release secondary to brain ischemia, and (iii) subgroup analysis of prognostic effects linked to the CRP dynamics in the first 7 days after MT.

### Imaging analysis

The volume of brain tissue necrosis at the time of admission was quantitatively estimated from CT perfusion (CTP) data [RAPID software (iSchemaView, Menlo Park, CA, USA)] (25). Acquisition of CTP is part of an in-house stroke neuroimaging standard operating procedure and was thus available for all patients, irrespective of the timing of symptom onset. The infarct core at admission was defined as the volumetric sum of voxels showcasing cerebral blood flow < 30% to the contralateral hemisphere. Alberta Stroke Program Early CT Score (ASPECTS) was automatically assessed from admission CT imaging (iSchemaView, Inc., Menlo Park, CA, USA) for patients with occlusion of the MCA.

### Statistical analysis

Continuous and categorical variables are given as mean and standard deviation (SD) and frequencies if not indicated otherwise. Variables were compared using the Mann–Whitney *U*-test and unpaired student *t-*test, as appropriate. Receiver operator characteristic (ROC) curve analysis was used to determine the association of continuously measured CRP levels and outcomes. The Youden index was subsequently determined to find optimal cutoff values for CRP levels. The relationship between outcomes and elevated CRP levels (defined cutoff of 5 mg/L according to in-house laboratory standards) was expressed as an odds ratio (OR), with a corresponding 95% confidence interval (CI) through logistic regression.

To correct for confounders, adjustments for the impact of age, sex, atrial fibrillation, hypertension, diabetes mellitus, estimated CTP infarct volume, premorbid mRS levels, and reperfusion success were made with multiple logistic regression. A moderation analysis was performed to check the interaction of the subgroups with/without atrial fibrillation on the association between CRP levels and outcomes under consideration of the abovementioned covariates (26).

Statistical analyses were performed using IBM SPSS Statistics version 28.0.0. for macOS (IBM, USA).

## Results

### Patient demographics and clinical data

Over the retrieval period, 1,012 patients were referred for MT of a LVO. Of these, 62 were lost to follow-up. Furthermore, 274 patients were either “wake-up” strokes or had an infection either pre-existing or developing within the first 7 days of in-clinic stay (mean diagnosis at 4.3 days post-MT) and were thus excluded, leading to a study collective of 676 patients at a mean age of 74 ± 13 years (48.5% men). Mean CRP levels at admission were 1.7 ± 3.2 mg/l and elevated in 313 patients (46.3%). Patients with elevated CRP levels experienced higher frequencies of atrial fibrillation (57.5 vs. 41.3%, *p* < 0.001), hypertension (64.9 vs. 60.6%, *p* = 0.01), and diabetes mellitus (26.2 vs. 16.8%, *p* = 0.003), as well as leukocyte counts (10.46 ± 6.79 G/l vs. 9.79 vs. 3.39 G/l, *p* < 0.001). Apart from this, both groups had similar clinical profiles (Table 1).

**Table 1:** Patient demographics for the study cohort and dichotomized according to CRP levels.

	**Subgroups**			
	**All**	**CRP** ≥**5 mg/l**	**CRP**<**5 mg/l**	***P***
	**(*****n*** = **676)**	**(*****n*** = **313)**	**(*****n*** = **363)**	
Age	73.87 ± 13.37	75.4 ± 13.2	72.5 ± 13.3	0.005
%Male	328 (48.5%)	152 (48.6%)	176 (48.5%)	0.98
NIHSS at admission	13 ± 6.76	13 ± 6.64	13 ± 6.87	0.92
Hypertension	439 (64.9%)	219 (70.0%)	220 (60.6%)	0.01
Diabetes	143 (21.2%)	82 (26.2%)	61 (16.8%)	0.003
Smoking^*^	157 (23.2%)	78 (24.9%)	79 (21.8%)	0.34
Hyperlipidemia	143(21.2%)	72 (23.0%)	71 (19.6%)	0.28
LLT	173 (25.6%)	90 (28.8%)	83 (22.9%)	0.08
Anticoagulation	195 (28.8%)	90(28.8%)	105 (28.9%)	0.98
Atrial fibrillation	330 (48.8%)	180 (57.5%)	150 (41.3%)	< 0.001
TOAST 1	94 (13.9%)	41 (13.1%)	53 (14.6%)	0.57
TOAST 2	324 (47.6%)	168 (53.4%)	155 (42.7%)	0.006
TOAST 3	0 (0%)	0 (0%)	1 (0%)	0.99
TOAST 4	42 (6.2%)	20 (6.4%)	22 (6.1%)	0.87
TOAST 5	216 (32.0%)	84 (26.9%)	132 (36.4%)	0.008
CRP levels (mg/l) (admission)	1.71 ± 3.15	31.0 ± 37.7	2.0 ± 1.5	< 0.001
Leukocytes (G/l) (admission)	10.46 ± 6.79	11.24 ± 9.27	9.79 ± 3.39	0.009
Time to admission (min)	55 ± 62	52 ± 41	58 ± 72	0.19
Time to reperfusion	191 ± 144	192 ± 138	189 ± 146	0.79
i.v. thrombolysis	289 (42.8%)	116 (37.1%)	173 (47.7%)	0.006
ASPECTS	4.97 ± 4.10	4.95 ± 4.00	5.0 ± 4.2	0.87
Infarct core at admission (ml)	20.2 ± 37.1	24.2 ± 41.2	17.3 ± 29.4	0.014
% mTICI 2b or better	590 (87.3%)	283 (90.4%)	307 (84.6%)	0.02

LLT, lipid-lowering therapy. ASPECTS are provided for patients with occlusion of the middle cerebral artery, only (*n* = 384). ^*^Current smoker. TOAST 1, large artery atherosclerosis; TOAST 2, cardioembolism; TOAST 3, small-vessel occlusion; TOAST 4, stroke of other determined etiology; TOAST 5, stroke of undetermined etiology.

### Stroke- and MT-related parameters

In descending frequency, the most commonly occluded vessels were middle cerebral artery (*n* = 384, 56.8%), distal segment of the internal carotid artery (*n* = 73, 10.8%), combination of >1 occluded intracranial vessel (*n* = 59, 8.7%), tandem occlusions (*n* = 53, 7.8%), basilar artery (*n* = 44, 6.5%), proximal segment of the internal carotid artery (*n* = 37, 5.5%), posterior cerebral artery (*n* = 11, 1.6%), anterior cerebral artery (*n* = 8, 1.2%), and the vertebral artery (*n* = 7, 1.0%). Symptom severity was moderate on average with mean NIHSS scores of 13 ± 6.8. Counting from the onset of clinical symptoms, the time to admission (52 ± 41 min vs. 58 ± 72 min, *p* = 0.19) and the time to reperfusion (191 ± 144 min vs. 189 ± 146 min, *p* = 0.79) were similar for both groups. I.v. thrombolysis was performed significantly less frequently in the cohort with elevated CRP levels (37.1 vs. 47.7%, *p* = 0.006). Moreover, patients with elevated CRP levels demonstrated larger estimated infarct cores on CTP (24.2 ± 41.2 ml vs. 17.3 ± 29.4 ml, *p* = 0.014) albeit comparable infarct areas as estimated by ASPECTS (4.95 ± 4.0 vs. 5.0 ± 4.2, *p* = 0.0.87) in the subgroup with MCA occlusion. Moreover, the thrombectomy results were better in patients whose initial CRP levels were elevated with 90.4 % (against 84.6%, *p* = 0.02) of final thrombectomy scores being mTICI 2b or better.

### Outcomes

**Figure 1:**
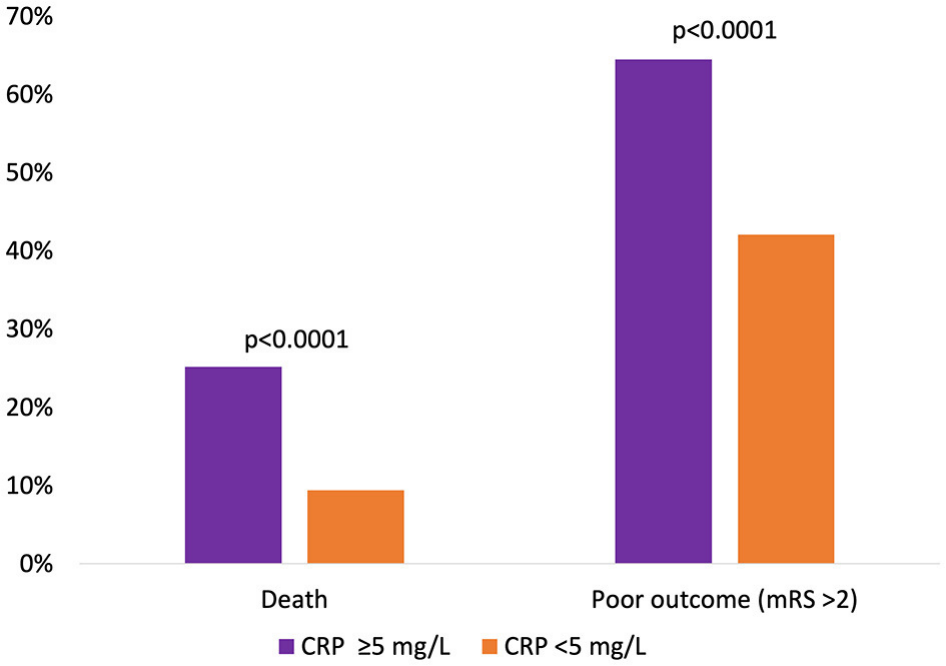
Relative occurrence of death or poor clinical outcome 90 days after mechanical thrombectomy, dichotomized according to initial CRP levels.

After 90 days of thrombectomy, a total of 113 patients (16.7%) were deceased with death occurring significantly more often in patients showcasing high-CRP levels *ad initio* (25.2 vs. 9.4%, *p* < 0.0001) (Figure 1). Median mRS after 90 days was 3 (IQR: 2; 5) and at a disadvantage in the high-CRP group with 4 (2; 6) vs. 2 (1; 4) (*p* < 0.0001). Brain hemorrhage up until the day of discharge, as verified by CT or MR imaging, was noted in a total of 15/460 patients (3.3%) for whom postprocedural imaging was available and at comparable rates in both groups (3.7 vs. 3.0%, *p* = 0.69). NIHSS values at discharge were available for 460/676 patients. A SNI at discharge could be noted in 206 of these patients. The frequency of SNI showed a trend to be higher in patients with normal initial CRP values (141/297, 47.5%) than in patients with elevated initial CRP values (65/163, 39.9%) (*p* = 0.11). Refer to Table 2 for a detailed outcome analysis.

**Table 2:** Occurrence of primary outcomes 90 days after mechanical thrombectomy, as well as secondary endpoint occurrence ICH and re-occlusion, as verified by CT or MRI, up until discharge.

**Primary outcomes**	**Subgroups**			
	**All**	**CRP** ≥**5 mg/l**	**CRP**<**5 mg/l**	***P***
	**(*****n*** = **676)**	**(*****n*** = **313)**	**(*****n*** = **363)**	
Mortality	113 (16.7%)	79 (25.2%)	34 (9.4%)	< 0.001
mRS	3 (2; 5)	4 (2; 6)	2 (1; 4)	< 0.001
Poor outcome (mRS > 2)	335 (49.6%)	213 (68.1%)	122 (33.6%)	< 0.001
**Secondary outcomes**	**Subgroups**			
	**All**	**CRP** ≥**5 mg/l**	**CRP**<**5 mg/l**	***P***
	**(*****n*** = **460)**	**(*****n*** = **163)**	**(*****n*** = **297)**	
SNI	206 (44.8%)	65 (39.9%)	141 (47.5)	0.11
ICH	15 (3.3%)	6 (3.7%)	9 (3.0%)	0.69
Re-occlusion	31 (6.7%)	14 (8.6%)	17 (5.7%)	0.24

mRS, modified Rankin scale; SNI, substantial neurological improvement; ICH, intracranial hemorrhage.

### Association of inflammatory markers with outcomes

**Figure 2:**
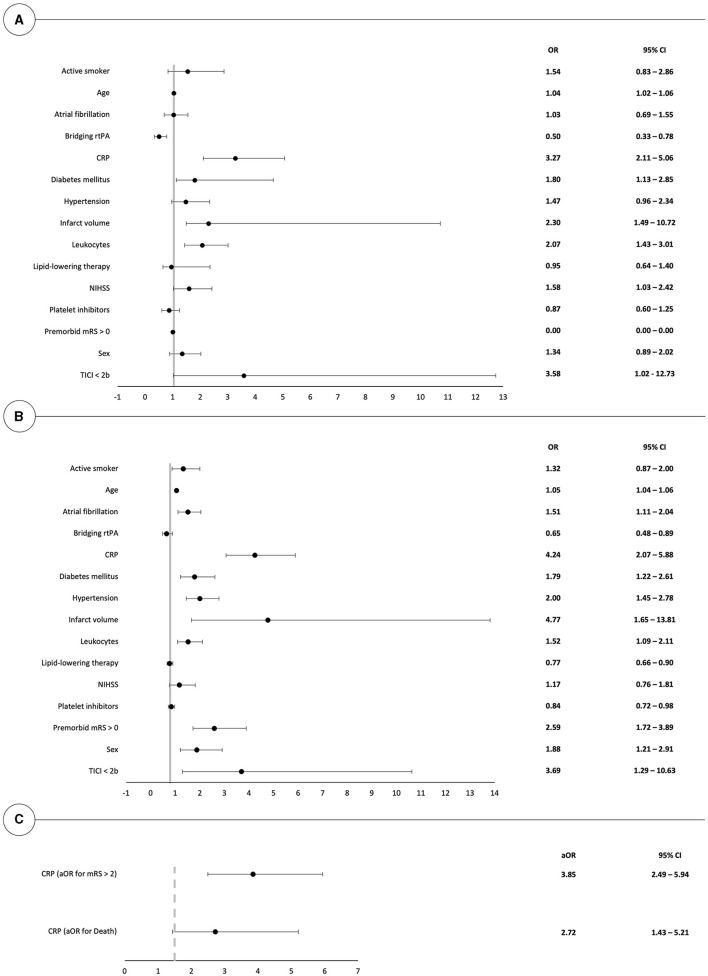
Forest plot illustrating the univariate risk for poor clinical outcome (mRS > 2) and death at 90 days after mechanical thrombectomy in **(A, B)**. **(C)** depicts the respective odds ratios in a multivariate model in patients with elevated CRP values. OR, odds ratio.

Odds ratios for death or poor outcome (mRS > 2) 90 days after thrombectomy have been calculated for all investigated parameters in univariate models, as shown in Figures 2A, B. In the decreasing order, CTP-estimated infarct volumes, elevated initial CRP levels, poor thrombectomy results (TICI < 2b), and pre-existing neurological impairments (mRS > 0) showed the highest association with poor outcomes after 90 days. On the other hand, poor thrombectomy outcomes (TICI < 2b), elevated CRP levels, CTP-estimated infarct volumes, and elevated leukocyte counts were most predictive for death at 90 days.

Due to the partial unavailability of confounding covariates, multivariate analysis and moderation analysis were calculated for a patient subset of *n* = 212.

Multivariate logistic regression, after correction for the effects of age, sex, atrial fibrillation, hypertension, diabetes mellitus, infarct volumes, pre-existing neurological impairments, and thrombectomy outcomes, highlights that initial CRP levels remained predictive for both mortality and poor mid-term outcome with respective odds ratios of 2.72 (95% CI: 1.43; 5.21) and 3.85 (95% CI: 2.49; 5.94), as shown in Figure 2C.

In a second step, the impact of the etiological subgroups with and without atrial fibrillation on the association between initial CRP levels and poor mid-term outcomes was tested in a moderation analysis under consideration of the abovementioned covariates. The conditional effect of initial CRP levels on poor outcomes remained significant for the atrial fibrillation group (*p* < 0.001). For the patient subgroup without atrial fibrillation, the conditional effect was lowered and lost its significance on the 5% level (*p* = 0.06), which implies that the importance of CRP levels for clinical outcomes is predominantly seen in patients with atrial fibrillation.

Multivariate logistic regression analyses for leukocyte counts lost their predictive power with respective odds ratios to predict mortality and poor mid-term outcomes of 0.92 (95% CI: 0.41, 2.05) and 0.73 (95% CI: 0.44, 1.23), respectively.

CRP values were further validated as a binary classifier for poor outcomes as a predictive model containing the above-referenced risk factors benefitted significantly from the inclusion of CRP measurements. As such, the AUC under the ROC increased from 0.74 (95% CI: 0.67, 0.81) to 0.81 (95% CI: 0.76, 0.87), as illustrated in Supplementary Figure 1. Calculation of the Youden Index yielded an optimal cutoff CRP level of 3.5 mg/l (sensitivity of 81.4% and specificity of 61%) and 8.5 mg/l (sensitivity of 69.0% and specificity of 69.0%) to predict poor outcomes and mortality, respectively.

### Endpoint-dependent CRP analysis

**Figure 3:**
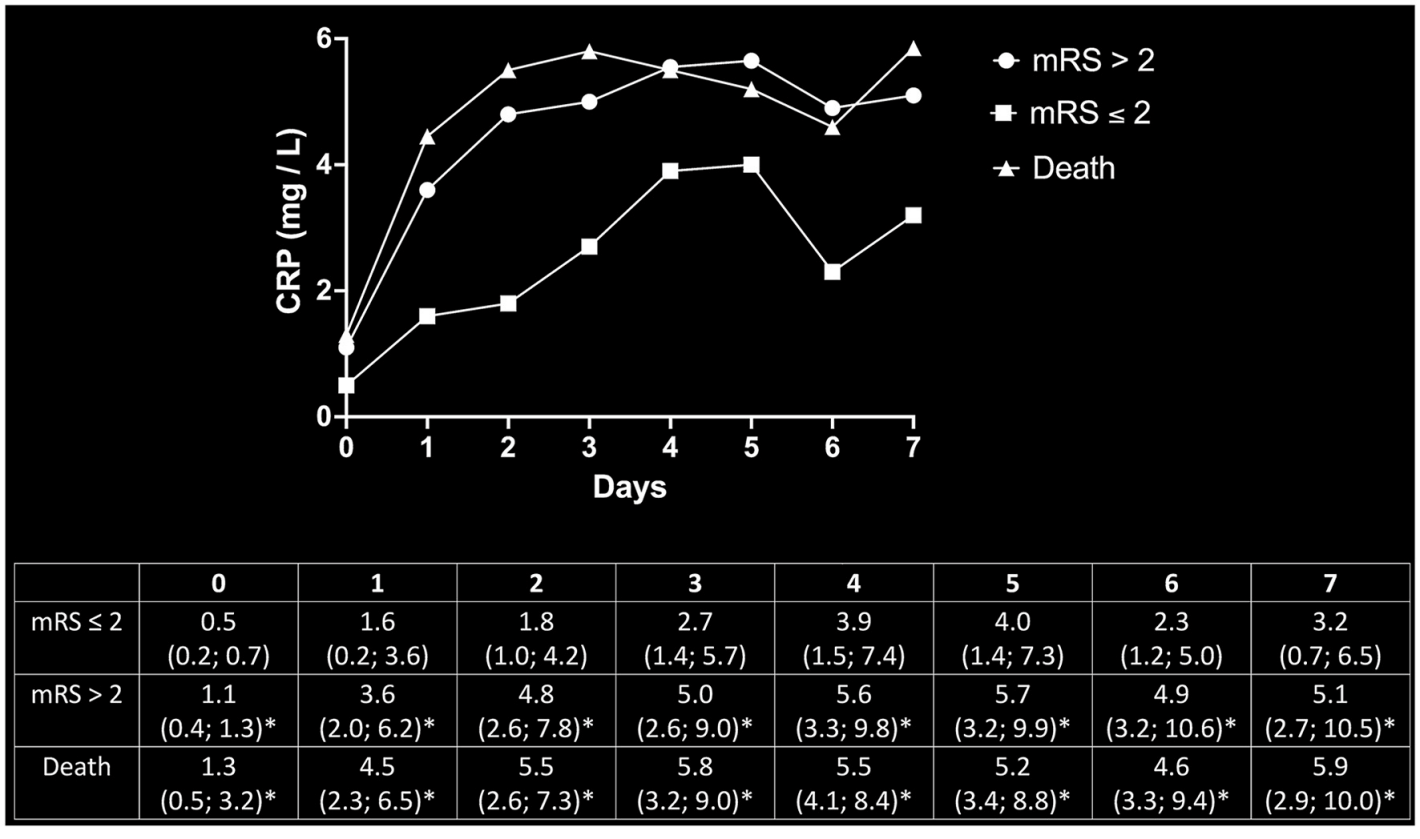
*Post-hoc* determination of CRP dynamics as a function of outcomes. Beyond the initial increase of inflammatory markers in patients with poor outcomes, the trajectory of CRP values continued to steepen over the consecutive 7 days after MT as compared to the cohort with favorable outcomes. The upper panel depicts the respective trajectories of CRP values for patients with good outcomes (mRS ≤ 2), poor outcomes (mRS > 2), and mortality. The corresponding median values and respective 95% CI are given in the lower panel. *Significantly above mRS ≤ 2 at the respective timepoint.

*Post-hoc* determination of CRP levels as a function of outcome showed that patients who reached the endpoint of mortality or mRS > 2 did not only have higher CRP levels *ad initio* but also experienced prolonged increases in CRP levels over the first 7 days post-MT. Endpoint-dependent CRP trajectories and values are given in Figure 3.

### Subgroup analysis for very-early inflammatory markers

In patients where initial blood sampling took place earlier than 4 h after symptom onset (*n* = 430), CRP levels were predictive for both poor outcomes [OR: 3.13 (95% CI, 2.06, 5.95)] and mortality [OR: 3.41 (95% CI, 2.11, 5.65)]. This effect remained intact in a multivariate model after correction for age, sex, atrial fibrillation, hypertension, diabetes mellitus, CTP-estimated infarct volume, premorbid mRS levels, NIHSS, and thrombectomy outcome for both poor clinical outcomes [OR: 2.59 (95% CI, 1.08, 6.41)] and mortality [OR: 2.94 (95% CI, 1.06, 9.07)]. Leukocyte counts, on the other hand, lost their predictive power for poor clinical outcomes after multivariate correction (Table 3).

**Table 3:** Subgroup analysis for very-early (< 4 h after symptom onset) CRP levels and leukocyte counts to predict mortality or poor clinical outcomes.

**Endpoint**	**Inflammatory marker**	**OR**	
		**Univariate**	**Multivariate**
MRS > 2	CRP	3.13 (2.06; 5.95)	2.59 (1.08; 6.41)
	Leukocytes	1.44 (0.94; 2.22)	1.51 (0.51; 4.60)
Mortality	CRP	3.41 (2.11; 5.65)	2.94 (1.06; 9.07)
	Leukocytes	2.26 (1.42; 3.59)	1.86 (1.07; 2.63)

## Discussion

The present study validates systemic inflammation markers, especially CRP levels, for predicting clinical outcomes in a large collective of endovascularly treated stroke patients. Our results show that (i) poor outcome and death occur significantly more often in stroke patients with elevated CRP levels before MT, particularly in patients with atrial fibrillation, (ii) these effects seem to be mediated by a systemic inflammatory environment and not as a response to brain tissue ischemia, and (iii) the ischemia-inflammation cascade in MT patients is more pronounced if CRP levels are elevated *ad initio*.

Evidence is growing that systemic inflammation has a pivotal role in many pathophysiological processes from cardiovascular disease to cancer progression and cognitive decline (27–29). CRP has already been identified as a sensible serum marker to predict adverse outcomes in cardiovascular events, and its levels are known to be influenced by genetic polymorphisms, an observation that could further deepen our understanding of the development and progression of vascular disease (30). CRP as an inflammatory marker has been associated with atrial fibrillation, including development, recurrence, and total burden, as well as associated with thromboembolic complications (21). It thus seems plausible that inflammation also influences the pathogenesis of ischemic stroke.

In this study, we are interested in finding out whether CRP levels in the acute phase of ischemic stroke can help explain the outcome disparities in patients with endovascularly treated LVO.

Our data show that CRP levels at admission are strongly and independently associated with an increased risk of mortality and poor clinical outcomes. In a broad, prospectively acquired collective of 676 patients, we thus noted that 70% of deaths at 90-day follow-up occurred in patients with initially elevated CRP levels. Moreover, after multivariate correction for confounding factors known to carry a negative prognostic value such as reperfusion failure or large volumes of estimated infarct cores, the relative risk of death or poor clinical outcomes remains significant if CRP levels were elevated *ad initio*. This observation held true even as patients with elevated CRP levels had smaller infarct volumes as well as more favorable thrombectomy results than those with no signs of systemic inflammation. The influence of etiology was also deliberated while atrial fibrillation was chosen as a covariate. Despite the known association between atrial fibrillation and elevated CRP levels, the inflammation marker has independently impacted outcomes. In a moderation analysis, it was shown that the importance of CRP levels for clinical outcomes is predominantly seen in patients with atrial fibrillation. This fits the known association of CRP levels in atrial fibrillation with burden and severity of thromboembolic events. It may have an impact on clinical practice as special focus should be placed on patients with cardioembolic stroke and elevated CRP levels regarding rehabilitation and secondary prevention.

Furthermore, our observation that CRP trajectories post-MT diverge in a way that initially high levels tend to further increase over the course of 7 days is of particular interest. One could speculate that CRP promotes a cascade where first-hit tissue damage promotes inflammatory mechanisms, again worsening tissue damage through known effects such as complement activation or T-cell platelet interaction (15, 31). Secondary burn progression in thermic injuries, where cell necrosis advances beyond the initial stress area in a time-delayed manner, could thus be a blueprint for some of the pathophysiological processes in brain ischemia. Moreover, the fact that injury in myocardial infarction has been mitigated by blocking CRP synthesis hints at the plausibility of such a cascade and causative role that CRP could have in the time-delayed cellular damage (32).

Validation of our findings in a multicentric setting could pave the way for interventional studies exploring CRP apheresis as a possible tool to break this ischemia-inflammation cascade in stroke patients (32, 33). Elegantly, this could potentially be achieved through intensified statin use, as one of the lipid-lowering agent properties is to dose-dependently reduce levels of not only LDL—known to also negatively impact the prognosis of ischemic stroke patients—but also CRP and other inflammatory markers (34). The hypothesis that in adjunct to reducing established cardio- and cerebrovascular risk factors, mitigating low-level systemic inflammation as a means of primary and secondary prevention gains momentum as a promising future avenue in stroke care (35).

Although CRP has been validated as an independent predictor of poor outcomes in patients with ICH (36), we found no correlation between early CRP levels and brain hemorrhage or repeated LVO after MT. At first sight, this may seem counterintuitive given the vast evidence that links low-level inflammation to endothelial dysfunction and might be due to the overall low rates of these secondary endpoints in our study cohort (37). Moreover, leukocyte counts on admission carried less prognostic weight as this marker reacts more slowly to a pro-inflammatory stimulus and might thus be less well suited as an early predictive marker.

Testing for CRP-related genetic polymorphisms would have provided a significant value to our analysis as this could have clarified if CRP is really the hallmark agent linked to poor outcomes after MT or just a surrogate marker for low-level systemic inflammation.

The study design in its very nature was retrospective with all inherent limitations. A further limitation is the monocentric study setting, calling for prospective multicentric studies to validate our findings, as well as the multivariate analysis with less power due to the partial unavailability of confounding variables. All-cause mortality after 90 days was chosen as an endpoint due to the common impossibility to determine a specific cause of death, especially in a cohort of elderly patients. It can, however, be assumed that the large cohort size flattens out potential inequalities in the rates of confounding co-morbidities among both groups. Body temperatures were not available although this metric might have provided further information on the potential onset of a latent infection during the clinical stay. Moreover, definite volumes of the infarcted tissue were not quantitatively assessed. Finally, patients with elevated CRP levels were on average 3 years older, with intuitive prognostic implications, a confounder mitigated by the multivariate correction we performed in our analysis.

To conclude, our study provides evidence for the negative prognostic value of acute-stage CRP levels in stroke patients treated via MT. In a set of more than 600 patients, we show that mid-term clinical outcomes and mortality are significantly and independently associated with initial CRP levels, particularly in patients suffering from atrial fibrillation. Our findings encourage the theory that mitigating low-level systemic inflammation could be a promising step to improve the prognosis of stroke patients. Based on this, data questions on acute vs. chronic and systemic inflammation should be further investigated in larger studies.

## Data availability statement

The datasets presented in this article are not readily available because of ethical and privacy restrictions. Requests to access the datasets should be directed to TF, tom.finck@tum.de.

## Ethics statement

The studies involving human participants were reviewed and approved by Local Ethics Committee. Written informed consent for participation was not required for this study in accordance with the national legislation and the institutional requirements.

## Author contributions

All authors listed have made a substantial, direct, and intellectual contribution to the work and approved it for publication.
