# Supplementary figures and images for: Inflammation in stroke: initial CRP levels can predict poor outcomes in endovascularly treated stroke patients
Source: Front Neurol. 2023 Jun 9;14:1167549. doi: 10.3389/fneur.2023.1167549 (PMC10289003; doi:10.3389/fneur.2023.1167549)

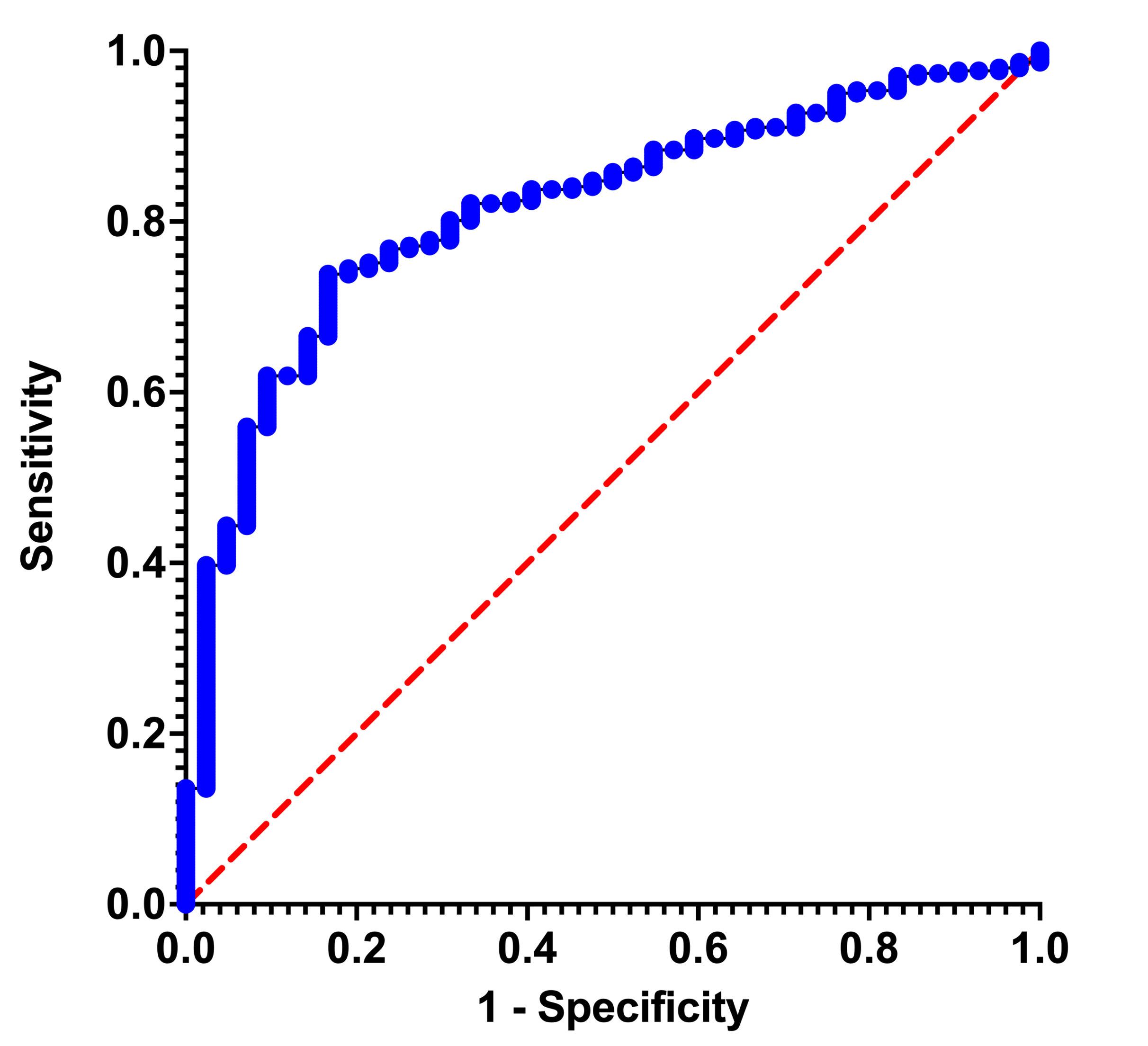

Supplement: Supplementary file 1 [file Image_1.png]
